# Small Bowel Perforation Caused by Uterine Iatrogenic Perforation Secondary to Endometrial Curettage: A Report of a Rare Case

**DOI:** 10.7759/cureus.91942

**Published:** 2025-09-09

**Authors:** Efthymia Thanasa, Anna Thanasa, Emmanouil M Xydias, Ioannis-Rafail Antoniou, Evangelos Kamaretsos, Gerasimos Kontogeorgis, Apostolos C Ziogas, Ioannis Paraoulakis, Dimitrios Papagoras, Ioannis Thanasas

**Affiliations:** 1 Department of Health Sciences, Medical School, Aristotle University of Thessaloniki, Thessaloniki, GRC; 2 Department of Obstetrics and Gynecology, EmbryoClinic IVF, Thessaloniki, GRC; 3 Department of Obstetrics and Gynecology, General Hospital of Trikala, Trikala, GRC; 4 Department of Obstetrics and Gynecology, University General Hospital "Attikon" Medical School, National and Kapodistrian University of Athens, Athens, GRC; 5 Department of Obstetrics and Gynecology, University of Thessaly, Larissa, GRC; 6 Department of Surgery, General Hospital of Trikala, Trikala, GRC

**Keywords:** case report, computed tomography, diagnostic curettage, enterectomy, fecal peritonitis, ileostomy, postmenopausal metrorrhagia, prognosis, small bowel perforation, uterine perforation

## Abstract

This report describes a rare case of small bowel perforation associated with iatrogenic uterine perforation during endometrial curettage, which was successfully treated despite delayed diagnosis. A 62-year-old patient, in septic condition and exhibiting symptoms of acute abdomen, was admitted to our hospital five days after undergoing diagnostic endometrial curettage due to postmenopausal metrorrhagia. Inflammatory markers were elevated. An emergency computed tomography (CT) scan revealed the presence of pneumoperitoneum and multiple fluid collections with air-fluid levels throughout the peritoneal cavity. Emergency laparotomy was performed, with severe fecal peritonitis secondary to small bowel perforation being observed. No primary uterine perforation site was confirmed intra-operatively, although histological examination of the endometrial curettage material confirmed the presence of smooth muscle tissue and debris of intestinal epithelium. Salvage surgery for neglected peritonitis was carried out, including enterectomy that involved the perforation site and creation of two ileostomies (end ileostomy and mucus fistula). The patient's worsening condition prompted admission to the intensive care unit (ICU), where she remained for 22 days and was subsequently transferred to the surgical ward, where she remained for another 15 days. She was discharged from the ward in good condition with instructions for follow-up assessment and scheduling of bowel restoration surgery. Based on the description of this rare case, the diagnostic approach and therapeutic options for this rare complication are discussed. Special emphasis is placed on the benefits of early diagnosis, which significantly contributes to ensuring the best prognostic outcome.

## Introduction

Diagnostic endometrial curettage constitutes a common method for endometrial tissue sampling and histopathological study of the endometrium, especially relevant during endometrial cancer investigation in postmenopausal patients with abnormal uterine bleeding [1]. It is a widely available diagnostic method, which, however, is more disadvantageous compared to hysteroscopy and pipelle endometrial biopsy, due to the requirement for general anesthesia and its higher complication rate [2]. Such complications primarily include uterine bleeding, but on rare occasions, they may also include uterine perforation, meaning severe injury of the uterine wall with loss of its integrity and communication of the uterine cavity with the peritoneal cavity [3].

Uterine perforation usually occurs at the fundus and is less frequent at the anterior wall and the cervix. The instrument most often involved is the sharp curette [4]. The rate of uterine perforation in patients undergoing diagnostic curettage for investigating postmenopausal vaginal bleeding is approximately 0.2% of cases [5]. Iatrogenic perforation of the small intestine during endometrial biopsy is an extremely rare secondary complication occurring after uterine perforation, but it is a potentially life-threatening condition, associated with pneumoperitoneum and severe peritonitis [6]. This is further exacerbated in cases of neglected patients and delayed diagnosis of the condition, leading to more challenging treatment, increased patient morbidity, and worse prognosis.

The iatrogenic cause of the condition usually guides the diagnostic process. Sudden loss of resistance or insertion of instruments beyond the expected uterine length during endometrial curettage or vacuum aspiration procedures should lead to elevated clinical suspicion of perforation [5]. Diagnosis is also guided by clinical symptomatology, such as persistent or worsening abdominal or pelvic pain, signs of acute abdomen (guarding, rebound tenderness, etc.), and signs of internal bleeding and shock (hypotension, tachycardia, etc.). Imaging modalities, such as vaginal ultrasound and computed tomography (CT), may help further guide diagnosis via the detection of free fluid, air, or active bleeding and thus confirm clinical suspicion of uterine perforation [5]. Treatment of uterine and intestinal perforation may be conservative, with antibiotics, fluid support, and close monitoring in select cases with only minor injuries to the organs in question. However, in the majority of cases, surgical intervention is required, which, depending on perforation site and severity, may be minor, involving suturing of the wall defect, or major with hysterectomy and segmental bowel resection at the site of the perforation being required to effectively address the condition [5,6].

This report describes one such case of small bowel rupture associated with iatrogenic uterine perforation during endometrial curettage, which was successfully treated despite the delayed diagnosis. Furthermore, the diagnostic approach and therapeutic options for this rare complication are subsequently discussed, with particular emphasis on the benefits of early diagnosis.

## Case presentation

A 62-year-old patient was admitted to the emergency department of the General Hospital of Trikala, Greece, in a septic condition and exhibiting acute abdominal symptoms. The patient first reported acute abdominal pain five days prior, after she underwent diagnostic endometrial curettage due to postmenopausal metrorrhagia. The procedure was performed at a private clinic, following a transvaginal ultrasound investigation for her metrorrhagia. Since then, as reported by the patient, the abdominal pain was constant and diffuse across the entire abdomen, mainly localized in the lower abdomen, with progressively worsening intensity. The pain was not accompanied by vomiting or other notable symptoms. Mild analgesic medications prescribed by her attending physician provided only slight and temporary relief from the pain during these past five days. The patient was overweight (BMI = 29.3). Her medical history was free from comorbidities, and no pre-existing chronic gastrointestinal conditions were reported.

On clinical examination, the patient was febrile (37.9°C) with signs of an acute abdomen in a septic setting. The abdomen was distended, firm, with signs of peritoneal irritation. Her blood pressure was low (80/50 mmHg), and her heart rate was elevated (113 beats/min). Blood sampling was performed for urgent laboratory tests (Table 1) and cultures. Intravenous administration of cefoxitin (Mefoxil®) with a loading dose of 2 g and maintenance dose of 2 g every eight hours via slow infusion was performed, and an emergency computed tomography (CT) scan was ordered.

**Table 1:** Laboratory tests of the patient upon arrival at the emergency department (ED) and during hospitalization in the intensive care unit (ICU) and the surgical department (SD). PD: post-operative day; Ht: hematocrit; Hb: hemoglobin; PLT: platelets; WBC: white blood cells; NEUT: neutrophils; CRP: C-reactive protein; APTT: activated partial thromboplastin time; INR: international normalized ratio; FIB: fibrinogen; Glu: glucose; Cr: creatinine; K+: potassium; Na+: sodium; Ca+: calcium; P: phosphorus; Mg: magnesium; TBIL: total bilirubin; DBIL: direct bilirubin; IDBIL: indirect bilirubin; SGOT: serum glutamic oxaloacetic transaminase; SGPT: serum glutamate pyruvate transaminase; AMY: amylase; TALB: total albumins; PCT: procalcitonin.

Laboratory tests	Day of presentation to the ED (preoperative)	Day of admission to the ICU (first PD)	During hospitalization in the ICU (10th PD)	Day of discharge from the ICU (22nd PD)	During hospitalization in the SD (27th PD)	Day of discharge from the SD (37th PD)	Normal laboratory values
Ht	38.2%	35.0%	41.1%	28.3%	36.8%	36.5%	37.7–49.7%
Hb	13.5 gr/dl	12.3 gr/dl	14.0 gr/dl	9.5 gr/dl	11.9 gr/dl	12.7 gr/dl	11.8–17.8 gr/dl
PLT	340x10^3^/ml	358x10^3^/ml	162x10^3^/ml	333x10^3^/ml	289x10^3^/ml	237x10^3^/ml	150–350x10^3^/ml
WBC	25.6x10^3^/ml	40.32x10^3^/ml	22.12x10^3^/ml	9.06x10^3^/ml	8.74x10^3^/ml	5.64x10^3^/ml	4–10.8x10^3^/ml
NEUT	92.6%	94.1%	90.1%	56.9%	58.4%	61.0%	40–75%
CRP	33.81 mg/dl	19.54 mg/dl	11.88 mg/dl	2.64 mg/dl	2.60 mg/dl	1.30 mg/dl	<0.7 mg/dl
APTT	23.1 sec	32.9.6 sec	31.6 sec	31.3 sec	29.1 sec	29.1 sec	24.0–35.0 sec
INR	1.13	1.22	1.30	1.25	1.06	1.02	0.8–1.2
FIB	327 mg/dl	396 mg/dl	175 mg/dl	215 mg/dl	227 mg/dl	239 mg/dl	200–400 mg/dl
Glu	134 mg/dl	210 mg/dl	130 mg/dl	83 mg/dl	84 mg/dl	101 mg/dl	75–115 mg/dl
Cr	1.0 mg/dl	0.92 mg/dl	0.57 mg/dl	0.39 mg/dl	0.53 mg/dl	0.85 mg/dl	0.40–1.10 mg/dl
Κ^+^	4.01 mmol/L	4.37 mmol/L	5.26 mmol/L	4.52 mmol/L	4.28 mmol/L	4.01 mmol/L	3.5–5.1 mmol/L
Να^+^	131.1 mmol/L	131.8 mmol/L	143.5 mmol/L	136.5 mmol/L	137.1 mmol/L	139.4 mmol/L	136–145 mmol/L
Ca^+^	-	7.2 mg/dl	8.3 mg/dl	9.1 mg/dl	9.3 mg/dl	-	8.1–10.4 mg/dl
P	-	4.3 mg/dl	-	3.9 mg/dl	-	-	2.6–4.5 mg/dl
Mg	-	1.91 mg/dl	-	1.48 mg/dl	-	-	1.6–2.3 mg/dl
TBIL	1.31 mg/dl	1.90 mg/dl	2.10 mg/dl	3.14 mg/dl	1.60 mg/dl	1.43 mg/dl	0.3–1.2 mg/dl
DBIL	-	1.03 mg/dl	0.86 mg/dl	1.56 mg/dl	0.56 mg/dl	0.55 mg/dl	0.0–0.5 mg/dl
INBIL	-	0.87 mg/dl	-	-	-	-	0.0–0.7 mg/dl
SGOT	52 IU/L	46 IU/L	91 IU/L	118 IU/L	158 IU/L	93 IU/L	5–33 IU/L
SGPT	34 IU/L	30 IU/L	79 IU/L	105 IU/L	234 IU/L	101 IU/L	10–37 IU/L
AMY	524 IU/L	199 IU/L	-	-	61 IU/L	-	30–118 IU/L
TALB		4.31 gr/dl	4.37 gr/dl	4.48 gr/dl	7.32 gr/dl	-	6.4–8.3 gr/dl
PCT	-	5.15 ng/mL	2.27 ng/mL	1.63 ng/mL	0.25 ng/mL	0.15 ng/mL	<0.1 ng/mL*
*: Elevated PCT levels are an indicator of severe infection and sepsis. More specifically: PCT <0.5 ng/mL, infection is not excluded but is not indicative of serious infection, >2 ng/mL is indicative of systemic bacterial infection or severe localized inflammation, >10 ng/mL is indicative of sepsis.							

**Figure 1:**
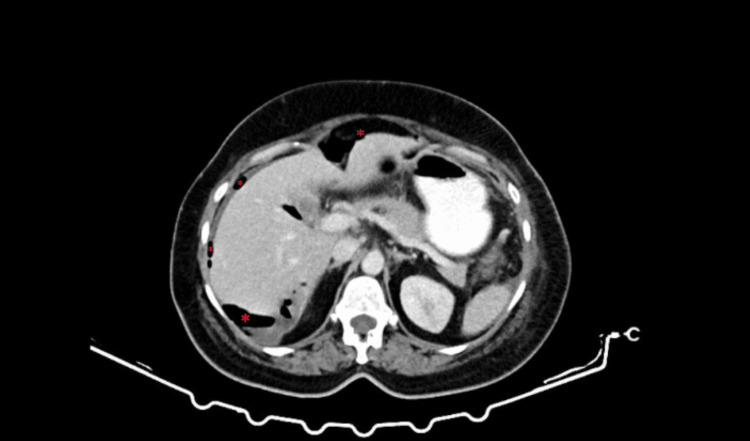
CT scan of the upper abdomen showing signs of fecal peritonitis and pneumoperitoneum caused by perforation of the small intestine. Red asterisks: air collections under the diaphragm (pneumoperitoneum).

**Figure 2:**
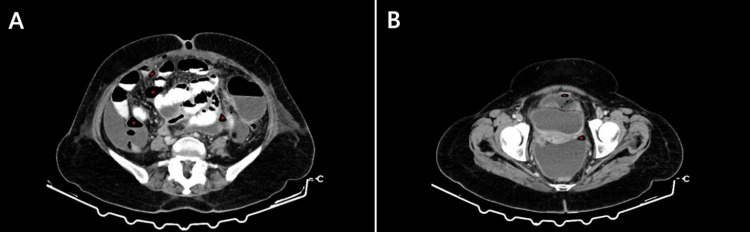
CT scan of the lower abdomen showing signs of peritonitis and encapsulated collections caused by perforation of the small intestine. Red asterisks: multiple encapsulated collections with air-fluid levels throughout the peritoneal cavity, located at (A): the paracolic regions bilaterally and (B): at the pouch of Douglas.

The scan revealed the presence of pneumoperitoneum with free air bubbles subdiaphragmatically and scattered in the peritoneal cavity (Figure 1). Additionally, multiple encapsulated, communicating collections with air-fluid levels were seen, located sub-hepatically, bilaterally paracolic between the intestinal loops, and in the posterior Douglas space (Figure 2). The largest collection had a craniocaudal diameter of approximately 13 cm.

**Figure 3:**
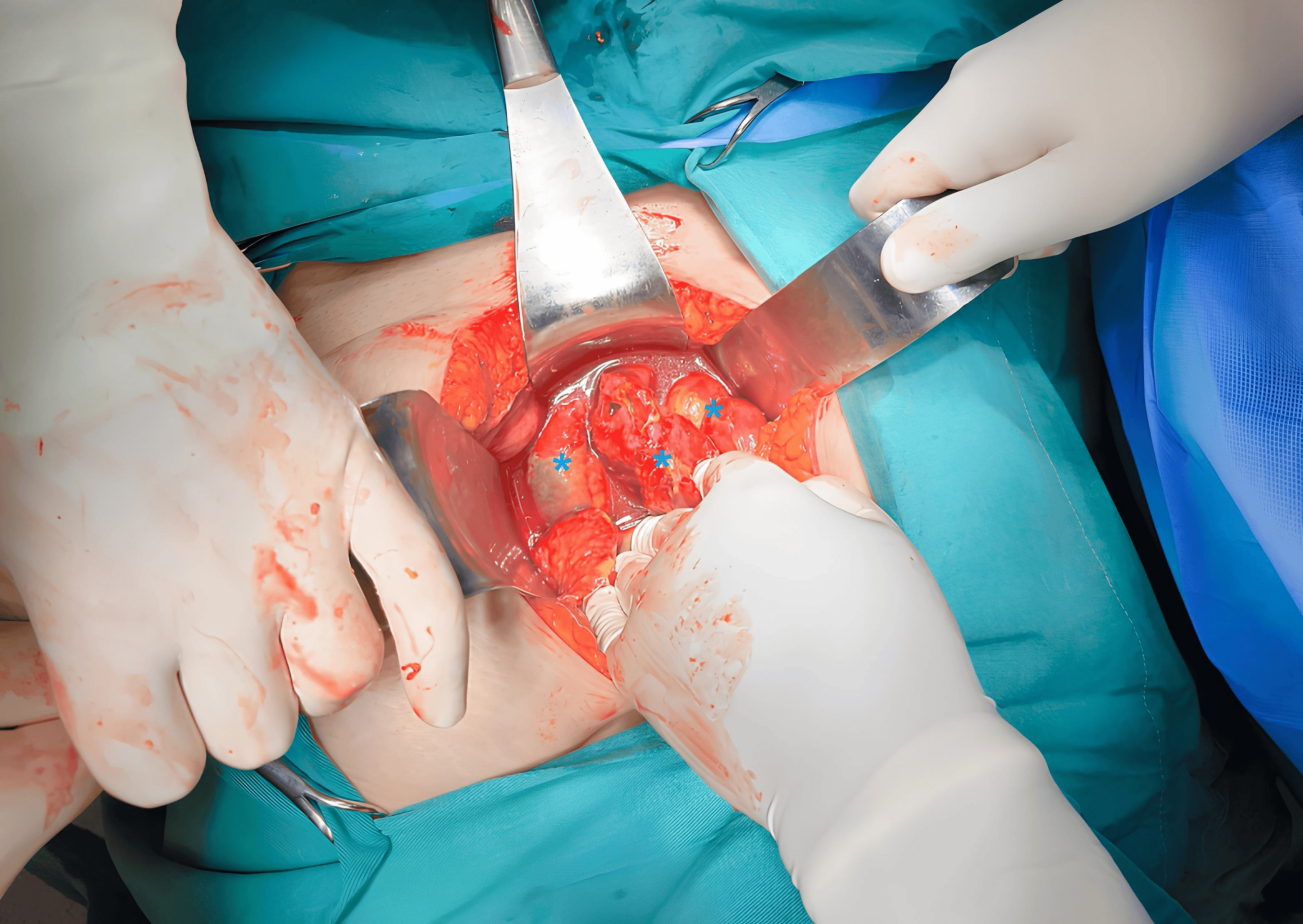
Intraoperative image of neglected fecal peritonitis. Βlue asterisks: small intestine loops with signs of sepsis and intense inflammation.

The clinical and laboratory findings indicated the presence of an acute abdomen and peritonitis, with imaging findings indicating that this was caused by abdominal or pelvic organ perforation (due to the presence of air in the abdominal cavity). Differential diagnosis included several conditions associated with perforation of intra-abdominal viscera, including diverticulitis, appendicitis, peptic ulcer rupture, ischemic colitis, etc. However, based on the recent history of endometrial curettage and the subsequent onset of symptoms, without any notable symptomatology pre-operatively, the working diagnosis of iatrogenic uterine perforation was made. Simultaneously, due to the severity of the clinical symptoms and laboratory findings, a possible secondary bowel rupture was also suspected, leading to an immediate laparotomy being performed. Intra-operatively, fecal material was identified within the peritoneal cavity, and severe fecal peritonitis due to small bowel rupture was confirmed. The image of severe neglected intra-abdominal sepsis, with firmly adherent pseudomembranes and signs of intense inflammation on the small bowel loops, was evident (Figure 3).

**Figure 4:**
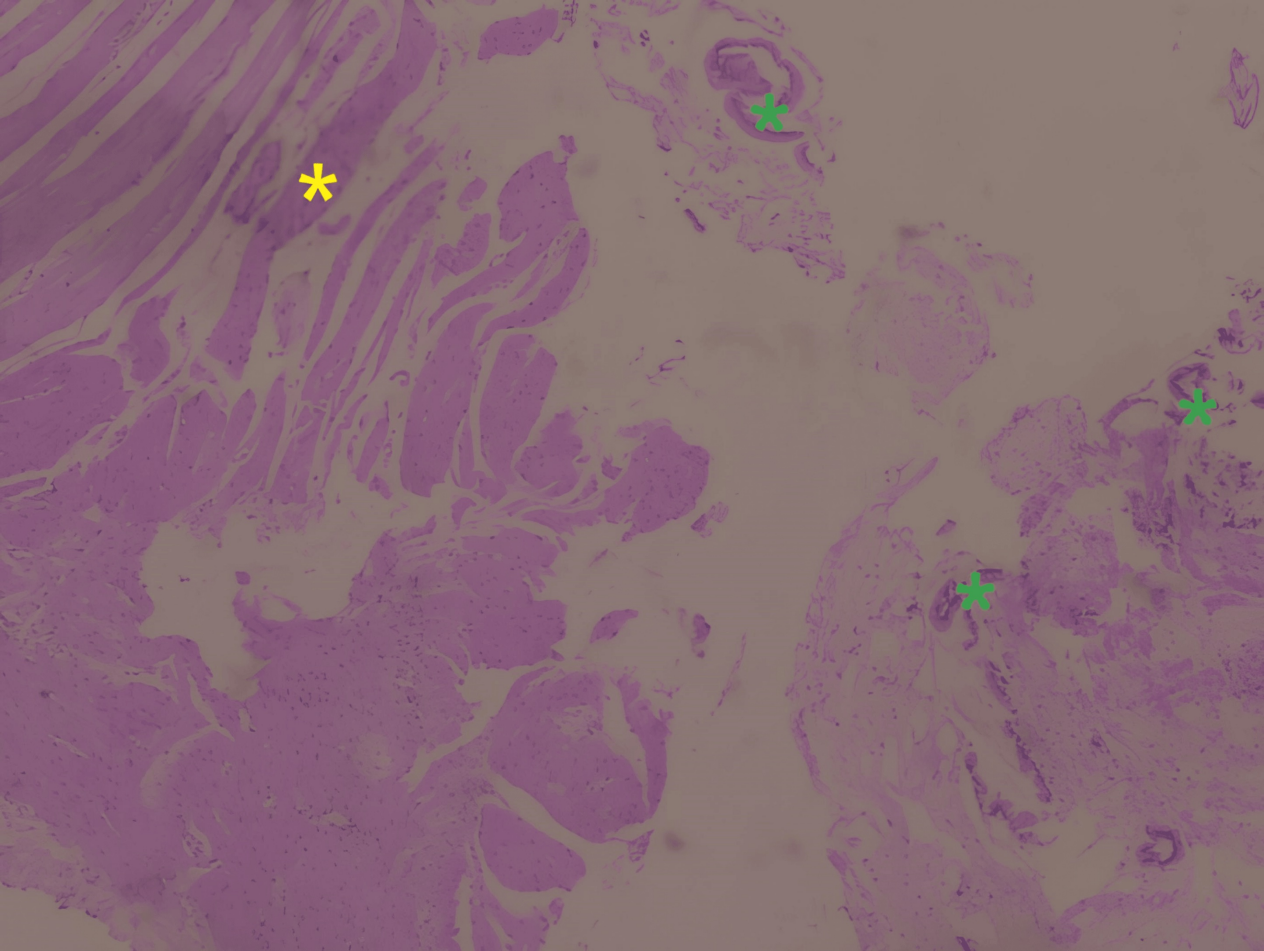
Histological image of the endometrial curettage sample. Yellow asterisk: smooth muscle fibers originating from the myometrium, green asterisks: intestinal epithelium debris (magnification, x10).

**Figure 5:**
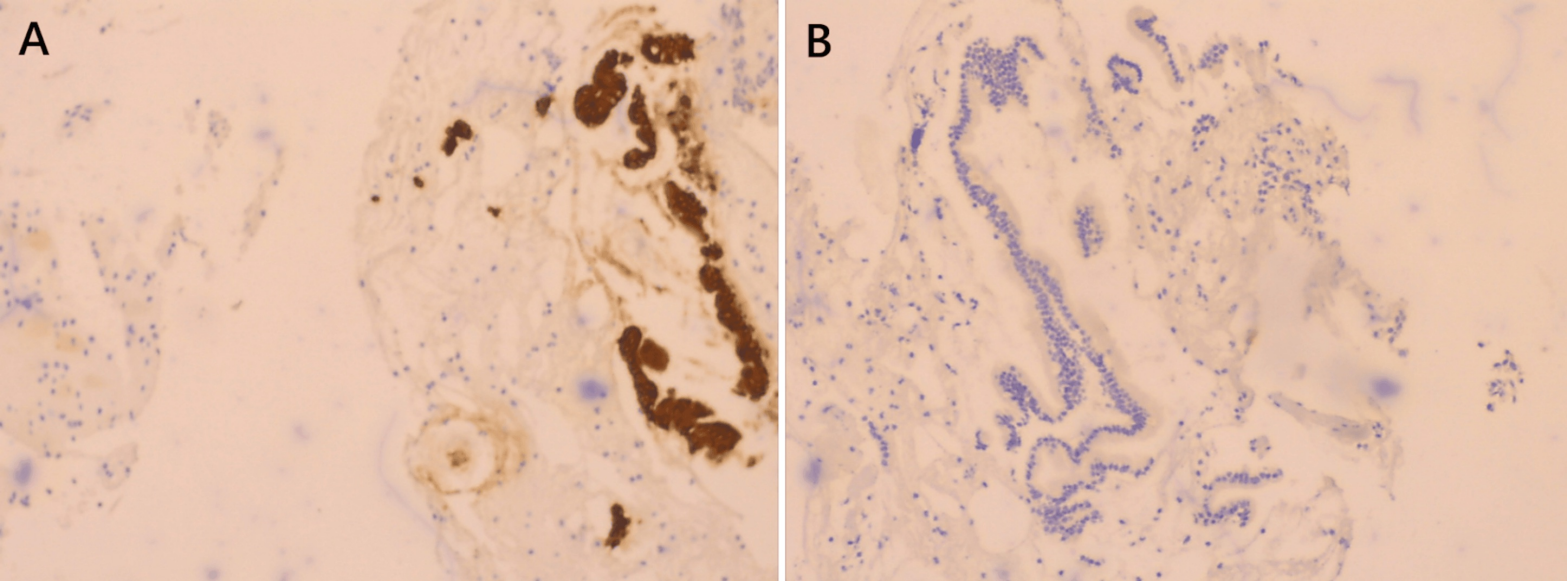
Immunohistochemical images of the endometrial curettage sample. (A) Cytokeratin 20 (CK20) positivity (magnification, x20), (B) estrogen receptors (ERs) negativity (magnification, x20).

The site of small bowel rupture was identified about 30 cm from the ileocecal valve and approximately 190 cm from the jejunum and had an approximate length of 5 cm. A small ecchymotic lesion without active bleeding was observed on the posterior uterine wall, at the level between the origin of the sacro-uterine ligaments, which seemed to be the most probable site of the uterine rupture; however, no macroscopically visible uterine wall defect was detected. At the time of surgery, since no visible uterine wall defect was observed and due to the patient's severe septic condition, the decision was made not to prolong surgery via a potentially unnecessary hysterectomy and instead focus on addressing the underlying cause of peritonitis, namely the intestinal perforation. Confirmation of primary uterine wall perforation was therefore not obtained during laparotomy; however, definitive diagnosis of both uterine and intestinal perforation was instead performed later, based on histopathological analysis of the original endometrial curettage specimens. The analysis revealed the presence of smooth muscle fibers, originating from the myometrium, and intestinal epithelium within the collected curettage specimen; findings that confirmed perforation of both the uterine and intestinal walls during initial curettage (Figures 4, 5).

The operation was subsequently taken over by the general surgery team, who decided to perform enterectomy that encompassed the rupture site and create two ileostomies (end ileostomy and mucus fistula), which was successful, but challenging due to abdominal obesity. After the conclusion of the surgery, the patient was transferred to the intensive care unit (ICU), where she remained in a critical condition (Table 1) for 22 days. During her ICU stay, she was supported with high doses of fluids, vasoconstrictive medications, transfusion of three units of blood (in addition to two units received intra-operatively), and broad-spectrum antibiotics including meropenem, metronidazole, colistin, and tigecycline. Subsequently, after being discharged from the ICU, the patient was transferred to the surgical ward, where she remained hospitalized for another 15 days. She was discharged from the surgical ward in good condition. Follow-up was scheduled at seven days post-discharge and included a multi-disciplinary team of gynecologists and general surgeons. The patient reported no gynecological symptoms, and physical and ultrasonographic examination revealed no abnormal findings. Assessment of the abdomen by general surgeons revealed no abnormal findings, and the ileostomies were functional and in good condition. Another follow-up examination took place 30 days post-operatively and verified that the ileostomies were still in good condition and that there were no other complications. At the time of writing, five months have elapsed since the patient's discharge, and follow-up by telephone has revealed no notable changes or new complications. The patient is scheduled to undergo ileostomy removal and bowel restoration surgery within the next months with an excellent prognosis.

## Discussion

In this report, we presented a rare complication of dilation and curettage, namely, uterine and subsequent bowel perforation, which was associated with increased patient morbidity, lengthy hospitalization, colostomy, and ICU admission. Uterine perforation is amongst the rarer complications of dilation and curettage, occurring during approximately 0.2% of operations in postmenopausal women [5,7], with the most common being hemorrhage. Uterine perforation, as a complication of curettage, may manifest with a plethora of clinical symptoms (depending on size and injury to other intra-peritoneal viscera, such as intestines and bladder); from asymptomatic hematomas and adhesions [8], to acute bleeding, hemoperitoneum, or bowel rupture, resulting in peritonitis [9]. It is estimated that the number and size of small bowel perforations do not seem to be related to the severity of the patient's clinical condition [10].

In our case, a single 5 cm bowel perforation was secondary to a very small initial uterine perforation, a phenomenon possibly caused by the angle of perforation and the relatively weaker constitution of the ileum's lumen compared to the uterine wall. Furthermore, in our patient, the perforation did not cause acute massive bleeding, only abdominal pain, erroneously attributed to surgery-related post-operative pain after endometrial curettage, despite its persistence for five days post-operatively. The lack of early clinical vigilance by the center performing the endometrial curettage procedure contributed significantly to delayed diagnosis, development of peritonitis, need for extensive surgery, and increased patient morbidity.

Early diagnosis of uterine and intestinal perforation could have been performed in this case by several imaging techniques. Abdominal X-ray is the first-line, readily available imaging technique that can reveal pneumoperitoneum with the presence of free air under the diaphragm, even in low-resource settings [6,11]. A CT scan is typically the initial imaging study used in modern practice for the investigation of the acute abdomen, being exceptional in detecting the presence of pneumoperitoneum, which confirms the occurrence of perforation [12,13]. The use of magnetic resonance imaging (MRI) is usually limited to clinically stable patients, and if selected, its application should not delay urgent intervention [14]. In our patient, her condition permitted only an emergency CT scan, whose findings, combined with the history of uterine curettage five days prior, were sufficient for the diagnosis of bowel perforation and peritonitis.

The diagnostic work-up followed during the management of this case is similar to other cases of uterine and bowel perforation described in the literature. Namely, the one by Samita Bhat et al. [15], who performed both CT and MRI scans during their investigation of a 57-year-old patient after curettage, since histopathological analysis of the collected tissues indicated the presence of intestinal epithelium, similar to the present case. In their case, however, no clinical symptoms were present post-operatively, and imaging studies indicated no abnormal findings with regard to the perforation. Surgery was performed nearly a month later and indicated no signs of peritonitis; however, firm adhesions and adherence of a bowel loop on the uterine wall were noted. That case of a silent perforation is in opposition to the observations made during the management of the present case, where the perforation caused fecal peritonitis and severe associated symptoms, later confirmed by CT. Use of CT was also employed in the case described by Dudhe et al., for whom hemoperitoneum and an abnormal uterine contour on CT were the primary indicators of perforation [16]. Lee et al. [17] utilized a simple abdominal X-ray scan as their first imaging approach, which did not show the presence of pneumoperitoneum, with follow-up examination via CT providing more conclusive evidence. In our case, the severity and urgency of the patient's symptoms prompted the use of CT directly, as the modality most likely to reliably identify the cause of acute abdomen and to confirm the clinical suspicion of uterine and bowel perforation. In a larger study by Mabula et al. [18], with 68 women with uterine and secondary bowel perforation due to induced abortion, the investigators primarily utilized clinical symptomatology (mainly abdominal pain, fever, and signs of peritonitis) and the known history of uterine surgery to diagnose the condition. Imaging studies were utilized to confirm the diagnosis of perforation. This is in alignment with the case presented here, as the known history of endometrial curettage and sudden onset of similar symptoms as those described in the aforementioned study guided diagnosis towards perforation, with CT being utilized for confirmation.

In the present case, it is likely that earlier referral of the patient to an imaging center by her attending physicians due to continued post-curettage pain would have led to earlier diagnosis of the perforation and more effective treatment with reduced morbidity. Confirmation of diagnosis was performed intra-operatively via laparotomy, although laparoscopy, a less invasive alternative, may have assisted in quicker patient recovery. However, even if laparoscopy was performed, the complexity of the underlying pathology would have mandated conversion to open surgery for treatment.

The treatment of uterine perforation is usually surgical and must be immediate when accompanied by hemodynamic instability of the patient. Conservative management with monitoring under antibiotic coverage is appropriate for select patients alone, with a stable condition and asymptomatic perforation [19], which was the case in the report by Samita Bhat et al. [15], who performed surgery 30 days after curettage and mostly to address the suspected endometrial carcinoma and not the perforation, due to the patient being asymptomatic. Even in cases of active bleeding, conservative treatment may be applied, such as in the case by Ziegler et al., who did succeed in conservatively managing uterine perforation with active bleeding after curettage using modified polysaccharide powder (4DryField®, PlantTec Medical GmbH, Lüneburg, Germany) [20]. In our case, this course of action was impossible, largely due to the unstable, worsening patient condition, which necessitated emergency exploratory laparotomy.

In most cases, management similarly involves surgical intervention, preferably via a laparoscopic approach, with suturing of the uterine wall or hysterectomy [21]. Hysterectomy was the treatment of choice in the report by Samita Bhat et al. [15], whereas Dudhe et al. opted for uterus-sparing wall repair [16]. A similarly less-radical surgical option was also selected for the case presented by Khan et al. [21], who treated a young patient with a large uterine wall perforation and omentum herniation via suturing and restoring the uterus's anatomy and integrity, thus preserving fertility. A more conservative surgical option was also preferred by Mabula et al., with 77.9% of cases being treated by uterine wall repair and only a minority of cases being treated by hysterectomy [18]. In our patient, since no visible rupture of the uterine wall was detected, potentially due to a small initial injury and the elapsed time until exploratory laparotomy, no surgical interventions on the uterus were performed. In an alternate clinical scenario, where the patient was brought in immediately after the endometrial curettage procedure that caused the perforation, diagnostic methods such as MRI and hysteroscopy may have helped identify the uterine wall defect, and repair could have been performed, in addition to bowel surgery. However, in the actual case, the patient's hospitalization was significantly delayed, causing her to develop severe peritonitis and entering surgery in an unstable condition, where the decision to prioritize bowel repair and peritoneal cleansing over performing exploratory hysteroscopy in order to confirm uterine perforation was made. Uterine perforation was ultimately microscopic at the time of surgery, confirmed by later histological and immunohistochemical examination of the endometrial cavity tissue collected during diagnostic curettage (Figures 4, 5).

With regard to the presence of secondary bowel perforation, emergency surgical intervention with either an open or laparoscopic approach is the only option [6,22]. The choice of surgical technique (primary repair or resection of the perforated small bowel segment with anastomosis) may depend on the number of perforations and the distance of the perforation site from the ileocecal valve. In cases where the small bowel perforation is singular, short, and far enough from the ileocecal valve, primary repair of the bowel wall is indicated. Regardless of the distance of the perforation site from the ileocecal valve, when the perforation is multiple or large, segmental bowel resection with anastomosis is indicated [10]. Segmental bowel resection with temporary ileostomy is only indicated in select patients with a poor prognosis [23]. Mabula et al. noted that the majority of patients were treated by bowel resection and anastomosis (86.8%) rather than perforation repair [18]. In our patient, although the perforation was singular and about 30 cm away from the ileocecal valve, the delay in diagnosis, which occurred five days after the traumatic bowel injury, the large length of the small bowel perforation (about 5 cm), and especially the severe neglected intra-abdominal sepsis (Figure 3) led to the decision to perform segmental bowel resection with a double temporary ileostomy rather than primary bowel wall repair.

The prognosis of uterine perforation is usually good. Exceptions include those patients in whom the diagnosis of uterine perforation is made with significant delay or is accompanied by injury to intra-abdominal organs [24]. In our patient, the delayed diagnosis of uterine perforation, made five days after the event, followed by secondary small bowel perforation and the development of severe neglected fecal peritonitis, resulted in a poor prognosis.

## Conclusions

Secondary perforation of the small intestine after uterine perforation as a complication of diagnostic curettage is a rare but particularly severe and life-threatening condition. Although this occurrence is rare, clinical suspicion of uterine perforation should remain elevated in patients with acute abdominal symptoms following diagnostic or therapeutic interventions in the uterus. In the present case, inattentive post-operative monitoring after curettage led to the need for extensive rescue surgery and admission to the ICU, with increased morbidity for the patient. While the observations made in this report are based on data from a single case and may lack applicability in different settings, they are indicative of the value of close post-operative monitoring of all patients undergoing endometrial diagnostic or therapeutic intervention, particularly in the case of blind dilation and curettage, facilitating early identification of any complications. In the presence of symptoms indicative of uterine and potentially bowel perforation, prompt confirmation via imaging and treatment are the most decisive factors affecting patient prognosis.

## References

[1] Dokara-Friedrich ML, Loeffler M, Shehaj I (2024). The clinical relevance of fractional curettage in the diagnostic management of primary endometrial cancer. Gynecol Obstet Invest.

[2] Luzarraga Aznar A, Canton R, Loren G (2025). Current challenges and emerging tools in endometrial cancer diagnosis. Int J Gynecol Cancer.

[3] Shen Y, Yang W, Liu J, Zhang Y (2023). Minimally invasive approaches for the early detection of endometrial cancer. Mol Cancer.

[4] Shakir F, Diab Y (2013). The perforated uterus. Obstet Gynaecol.

[5] Zorilă GL, Căpitănescu RG, Drăgușin RC (2023). Uterine perforation as a complication of the intrauterine procedures causing omentum incarceration: a review. Diagnostics (Basel).

[6] Vecchio R, Marchese S, Leanza V, Leanza A, Intagliata E (2015). Totally laparoscopic repair of an ileal and uterine iatrogenic perforation secondary to endometrial curettage. Int Surg.

[7] Vitale SG, Buzzaccarini G, Riemma G (2023). Endometrial biopsy: indications, techniques and recommendations. An evidence-based guideline for clinical practice. J Gynecol Obstet Hum Reprod.

[8] Hirschelmann A, Tchartchian G, Wallwiener M, Hackethal A, De Wilde RL (2012). A review of the problematic adhesion prophylaxis in gynaecological surgery. Arch Gynecol Obstet.

[9] Munro MG, Christianson LA (2015). Complications of hysteroscopic and uterine resectoscopic surgery. Clin Obstet Gynecol.

[10] Caronna R, Boukari AK, Zaongo D (2013). Comparative analysis of primary repair vs resection and anastomosis, with laparostomy, in management of typhoid intestinal perforation: results of a rural hospital in northwestern Benin. BMC Gastroenterol.

[11] Maebayashi A, Kato K, Hayashi N, Nagaishi M, Kawana K (2022). Importance of abdominal X-ray to confirm the position of levonorgestrel-releasing intrauterine system: a case report. World J Clin Cases.

[12] Hines J, Rosenblat J, Duncan DR, Friedman B, Katz DS (2013). Perforation of the mesenteric small bowel: etiologies and CT findings. Emerg Radiol.

[13] Jiang L, Wu J, Feng X (2019). The value of ultrasound in diagnosis of pneumoperitoneum in emergent or critical conditions: a meta-analysis. Hong Kong J Emerg Med.

[14] Aboughalia H, Basavalingu D, Revzin MV, Sienas LE, Katz DS, Moshiri M (2021). Imaging evaluation of uterine perforation and rupture. Abdom Radiol (NY).

[15] Samita Bhat K, Ahuja VK, Somashekhar SP (2018). Unusual bowel perforation following dilatation and curettage in a case of endometrial cancer. Indian J Gynecol Oncol.

[16] Dudhe SS, Waghulkar S, Mishra GV, Parihar P, Nimodia D (2024). A rare occurrence of uterine perforation following the dilation and curettage for missed abortion. Cureus.

[17] Lee MR, Kim MJ, Jeon HJ (2012). Two cases of intestinal perforation during dilatation and curettage in postpartum. Korean J Obstet Gynecol.

[18] Mabula JB, Chalya PL, Mchembe MD (2012). Bowel perforation secondary to illegally induced abortion: a tertiary hospital experience in Tanzania. World J Emerg Surg.

[19] Tchuenkam LW, Mbonda AN, Tochie JN (2021). Transvaginal strangulated bowel evisceration through uterine perforation due to unsafe abortion: a case report and literature review. BMC Womens Health.

[20] Ziegler N, Korell M, Herrmann A (2016). Uterine perforation following a fractional curettage successfully treated with the modified polysaccharide 4DryField® PH: a case report. J Med Case Rep.

[21] Khan IA, Shahi V, MaliK N (2023). Omental herniation through uterine perforation due to unsafe abortion: a case report. J Clin Diagn Res.

[22] Driák D, Sehnal B, Jarošová L (2022). Uterine perforation during intrauterine procedures and its management. Ceska Gynekol.

[23] Jain BK, Arora H, Srivastava UK (2010). Insight into the management of non-traumatic perforation of the small intestine. J Infect Dev Ctries.

[24] Heinemann K, Reed S, Moehner S, Minh TD (2015). Risk of uterine perforation with levonorgestrel-releasing and copper intrauterine devices in the European Active Surveillance Study on Intrauterine Devices. Contraception.

